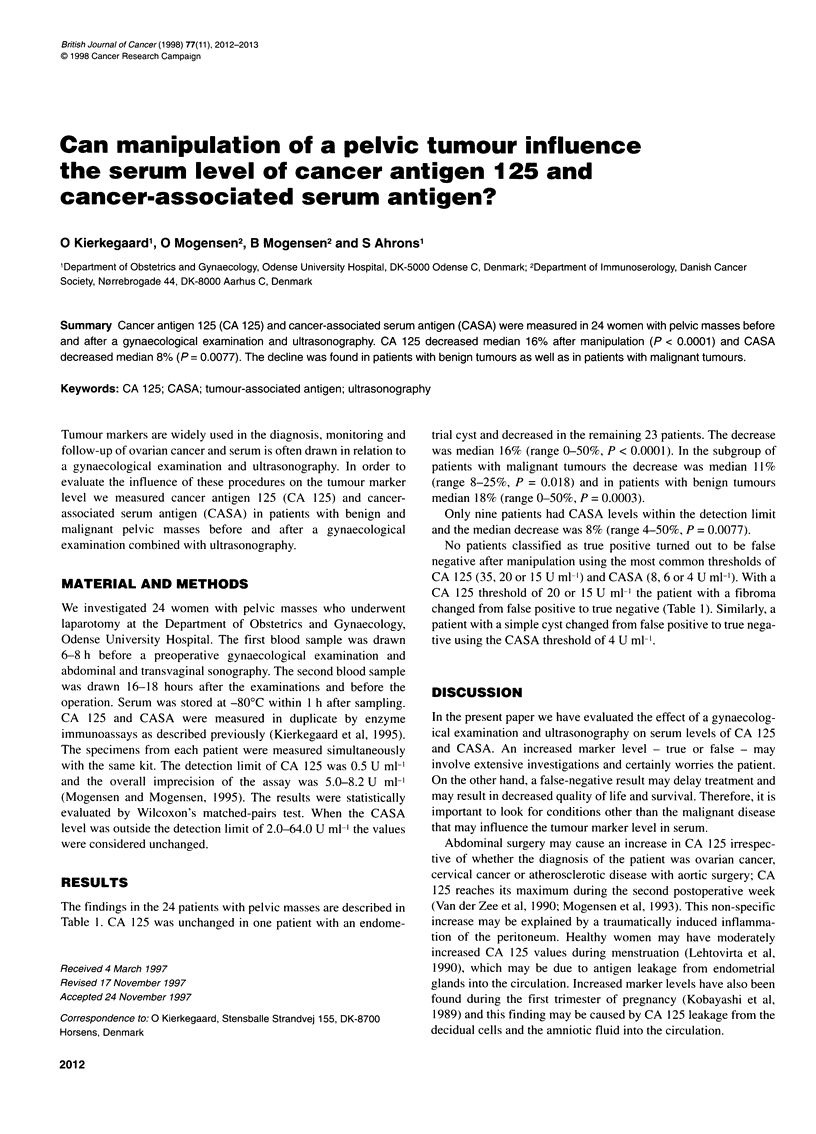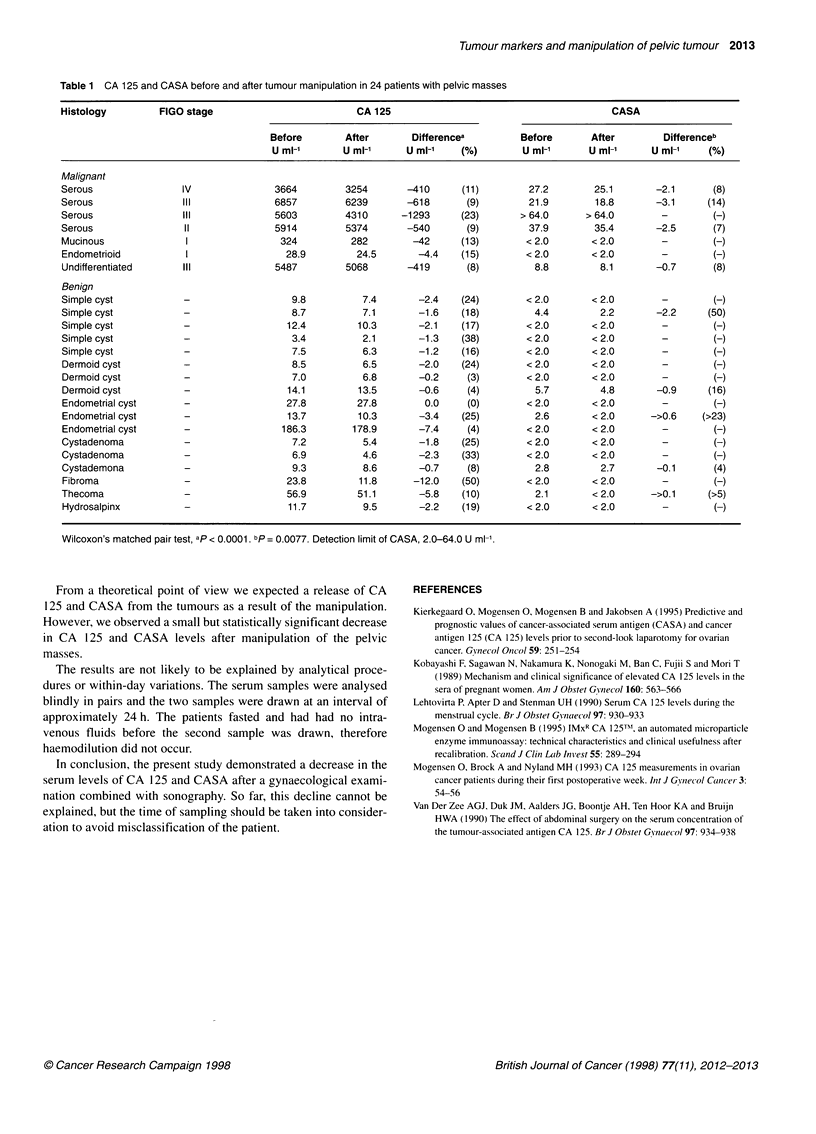# Can manipulation of a pelvic tumour influence the serum level of cancer antigen 125 and cancer-associated serum antigen?

**DOI:** 10.1038/bjc.1998.334

**Published:** 1998-06

**Authors:** O. Kiekegaard, O. Mogensen, B. Mogensen, S. Ahrons

**Affiliations:** Department of Obstetrics and Gynaecology, Odense University Hospital, Odense C, Denmark.

## Abstract

Cancer antigen 125 (CA 125) and cancer-associated serum antigen (CASA) were measured in 24 women with pelvic masses before and after a gynaecological examination and ultrasonography. CA 125 decreased median 16% after manipulation (P < 0.0001) and CASA decreased median 8% (P = 0.0077). The decline was found in patients with benign tumours as well as in patients with malignant tumours.


British Journal of Cancer (1998) 77(11), 2012–2013

© 1998 Cancer Research Campaign

### Can manipulation of a pelvic tumour influence the serum level of cancer antigen 125 and cancer-associated serum antigen?

**O Kierkegaard[1], O Mogensen[2], B Mogensen[2] and S Ahrons[1]**

[1]Department of Obstetrics and Gynaecology, Odense University Hospital, DK-5000 Odense C, Denmark; [2]Department of Immunoserology, Danish Cancer Society, Nørrebrogade 44, DK-8000 Aarhus C, Denmark

**Summary** Cancer antigen 125 (CA 125) and cancer-associated serum antigen (CASA) were measured in 24 women with pelvic masses before and after a gynaecological examination and ultrasonography. CA 125 decreased median 16% after manipulation ($P < 0.0001$) and CASA decreased median 8% ($P = 0.0077$). The decline was found in patients with benign tumours as well as in patients with malignant tumours.

**Keywords:** CA 125; CASA; tumour-associated antigen; ultrasonography

Tumour markers are widely used in the diagnosis, monitoring and follow-up of ovarian cancer and serum is often drawn in relation to a gynaecological examination and ultrasonography. In order to evaluate the influence of these procedures on the tumour marker level we measured cancer antigen 125 (CA 125) and cancer-associated serum antigen (CASA) in patients with benign and malignant pelvic masses before and after a gynaecological examination combined with ultrasonography.

### MATERIAL AND METHODS

We investigated 24 women with pelvic masses who underwent laparotomy at the Department of Obstetrics and Gynaecology, Odense University Hospital. The first blood sample was drawn 6–8 h before a preoperative gynaecological examination and abdominal and transvaginal sonography. The second blood sample was drawn 16–18 hours after the examinations and before the operation. Serum was stored at –80°C within 1 h after sampling. CA 125 and CASA were measured in duplicate by enzyme immunoassays as described previously (Kierkegaard et al, 1995). The specimens from each patient were measured simultaneously with the same kit. The detection limit of CA 125 was 0.5 U $ml^{-1}$ and the overall imprecision of the assay was 5.0–8.2 U $ml^{-1}$ (Mogensen and Mogensen, 1995). The results were statistically evaluated by Wilcoxon's matched-pairs test. When the CASA level was outside the detection limit of 2.0–64.0 U $ml^{-1}$ the values were considered unchanged.

### RESULTS

The findings in the 24 patients with pelvic masses are described in Table 1. CA 125 was unchanged in one patient with an endometrial cyst and decreased in the remaining 23 patients. The decrease was median 16% (range 0–50%, $P < 0.0001$). In the subgroup of patients with malignant tumours the decrease was median 11% (range 8–25%, $P = 0.018$) and in patients with benign tumours median 18% (range 0–50%, $P = 0.0003$).

Only nine patients had CASA levels within the detection limit and the median decrease was 8% (range 4–50%, $P = 0.0077$).

No patients classified as true positive turned out to be false negative after manipulation using the most common thresholds of CA 125 (35, 20 or 15 U $ml^{-1}$) and CASA (8, 6 or 4 U $ml^{-1}$). With a CA 125 threshold of 20 or 15 U $ml^{-1}$ the patient with a fibroma changed from false positive to true negative (Table 1). Similarly, a patient with a simple cyst changed from false positive to true negative using the CASA threshold of 4 U $ml^{-1}$.

### DISCUSSION

In the present paper we have evaluated the effect of a gynaecological examination and ultrasonography on serum levels of CA 125 and CASA. An increased marker level – true or false – may involve extensive investigations and certainly worries the patient. On the other hand, a false-negative result may delay treatment and may result in decreased quality of life and survival. Therefore, it is important to look for conditions other than the malignant disease that may influence the tumour marker level in serum.

Abdominal surgery may cause an increase in CA 125 irrespective of whether the diagnosis of the patient was ovarian cancer, cervical cancer or atherosclerotic disease with aortic surgery; CA 125 reaches its maximum during the second postoperative week (Van der Zee et al, 1990; Mogensen et al, 1993). This non-specific increase may be explained by a traumatically induced inflammation of the peritoneum. Healthy women may have moderately increased CA 125 values during menstruation (Lehtovirta et al, 1990), which may be due to antigen leakage from endometrial glands into the circulation. Increased marker levels have also been found during the first trimester of pregnancy (Kobayashi et al, 1989) and this finding may be caused by CA 125 leakage from the decidual cells and the amniotic fluid into the circulation.

*Received 4 March 1997*
*Revised 17 November 1997*
*Accepted 24 November 1997*

*Correspondence to:* O Kierkegaard, Stensballe Strandvej 155, DK-8700 Horsens, Denmark

**Table 1** CA 125 and CASA before and after tumour manipulation in 24 patients with pelvic masses

| Histology | FIGO stage | CA 125 | | | | CASA | | | |
|---|---|---|---|---|---|---|---|---|---|
| | | Before U ml⁻¹ | After U ml⁻¹ | Difference[a] U ml⁻¹ | (%) | Before U ml⁻¹ | After U ml⁻¹ | Difference[b] U ml⁻¹ | (%) |
| *Malignant* | | | | | | | | | |
| Serous | IV | 3664 | 3254 | –410 | (11) | 27.2 | 25.1 | –2.1 | (8) |
| Serous | III | 6857 | 6239 | –618 | (9) | 21.9 | 18.8 | –3.1 | (14) |
| Serous | III | 5603 | 4310 | –1293 | (23) | > 64.0 | > 64.0 | – | (–) |
| Serous | II | 5914 | 5374 | –540 | (9) | 37.9 | 35.4 | –2.5 | (7) |
| Mucinous | I | 324 | 282 | –42 | (13) | < 2.0 | < 2.0 | – | (–) |
| Endometrioid | I | 28.9 | 24.5 | –4.4 | (15) | < 2.0 | < 2.0 | – | (–) |
| Undifferentiated | III | 5487 | 5068 | –419 | (8) | 8.8 | 8.1 | –0.7 | (8) |
| *Benign* | | | | | | | | | |
| Simple cyst | – | 9.8 | 7.4 | –2.4 | (24) | < 2.0 | < 2.0 | – | (–) |
| Simple cyst | – | 8.7 | 7.1 | –1.6 | (18) | 4.4 | 2.2 | –2.2 | (50) |
| Simple cyst | – | 12.4 | 10.3 | –2.1 | (17) | < 2.0 | < 2.0 | – | (–) |
| Simple cyst | – | 3.4 | 2.1 | –1.3 | (38) | < 2.0 | < 2.0 | – | (–) |
| Simple cyst | – | 7.5 | 6.3 | –1.2 | (16) | < 2.0 | < 2.0 | – | (–) |
| Dermoid cyst | – | 8.5 | 6.5 | –2.0 | (24) | < 2.0 | < 2.0 | – | (–) |
| Dermoid cyst | – | 7.0 | 6.8 | –0.2 | (3) | < 2.0 | < 2.0 | – | (–) |
| Dermoid cyst | – | 14.1 | 13.5 | –0.6 | (4) | 5.7 | 4.8 | –0.9 | (16) |
| Endometrial cyst | – | 27.8 | 27.8 | 0.0 | (0) | < 2.0 | < 2.0 | – | (–) |
| Endometrial cyst | – | 13.7 | 10.3 | –3.4 | (25) | 2.6 | < 2.0 | –>0.6 | (>23) |
| Endometrial cyst | – | 186.3 | 178.9 | –7.4 | (4) | < 2.0 | < 2.0 | – | (–) |
| Cystadenoma | – | 7.2 | 5.4 | –1.8 | (25) | < 2.0 | < 2.0 | – | (–) |
| Cystadenoma | – | 6.9 | 4.6 | –2.3 | (33) | < 2.0 | < 2.0 | – | (–) |
| Cystademona | – | 9.3 | 8.6 | –0.7 | (8) | 2.8 | 2.7 | –0.1 | (4) |
| Fibroma | – | 23.8 | 11.8 | –12.0 | (50) | < 2.0 | < 2.0 | – | (–) |
| Thecoma | – | 56.9 | 51.1 | –5.8 | (10) | 2.1 | < 2.0 | –>0.1 | (>5) |
| Hydrosalpinx | – | 11.7 | 9.5 | –2.2 | (19) | < 2.0 | < 2.0 | – | (–) |

Wilcoxon's matched pair test, [a]$P < 0.0001$. [b]$P = 0.0077$. Detection limit of CASA, 2.0–64.0 U ml⁻¹.

From a theoretical point of view we expected a release of CA 125 and CASA from the tumours as a result of the manipulation. However, we observed a small but statistically significant decrease in CA 125 and CASA levels after manipulation of the pelvic masses.

The results are not likely to be explained by analytical procedures or within-day variations. The serum samples were analysed blindly in pairs and the two samples were drawn at an interval of approximately 24 h. The patients fasted and had had no intravenous fluids before the second sample was drawn, therefore haemodilution did not occur.

In conclusion, the present study demonstrated a decrease in the serum levels of CA 125 and CASA after a gynaecological examination combined with sonography. So far, this decline cannot be explained, but the time of sampling should be taken into consideration to avoid misclassification of the patient.

### REFERENCES

Kierkegaard O, Mogensen O, Mogensen B and Jakobsen A (1995) Predictive and prognostic values of cancer-associated serum antigen (CASA) and cancer antigen 125 (CA 125) levels prior to second-look laparotomy for ovarian cancer. *Gynecol Oncol* **59**: 251–254

Kobayashi F, Sagawan N, Nakamura K, Nonogaki M, Ban C, Fujii S and Mori T (1989) Mechanism and clinical significance of elevated CA 125 levels in the sera of pregnant women. *Am J Obstet Gynecol* **160**: 563–566

Lehtovirta P, Apter D and Stenman UH (1990) Serum CA 125 levels during the menstrual cycle. *Br J Obstet Gynaecol* **97**: 930–933

Mogensen O and Mogensen B (1995) IMx[R] CA 125[TM], an automated microparticle enzyme immunoassay: technical characteristics and clinical usefulness after recalibration. *Scand J Clin Lab Invest* **55**: 289–294

Mogensen O, Brock A and Nyland MH (1993) CA 125 measurements in ovarian cancer patients during their first postoperative week. *Int J Gynecol Cancer* **3**: 54–56

Van Der Zee AGJ, Duk JM, Aalders JG, Boontje AH, Ten Hoor KA and Bruijn HWA (1990) The effect of abdominal surgery on the serum concentration of the tumour-associated antigen CA 125. *Br J Obstet Gynaecol* **97**: 934–938

*© Cancer Research Campaign 1998*